# Effectiveness of The Milan System for Reporting Salivary Gland Cytology: A 5-Year Retrospective Review 

**DOI:** 10.30699/ijp.2024.2021304.3249

**Published:** 2025-01-10

**Authors:** Afrooz Arashloo, Hana Saffar, Maryam Lotfi, Fereshteh Ameli

**Affiliations:** 1 *Department of Pathology, Cancer Institute, Imam Khomeini Hospital Complex, Tehran University of Medical Science, Tehran, Iran*; 2 *Department Pathology, School of Medicine Amir Alam Hospital Tehran University of Medical Sciences, Tehran, Iran*; 3 *Department of Pathology, School of Medicine, Maternal, Fetal and Neonatal Research Center, Family Health Research Institute, Imam Khomeini Hospital Complex, Tehran University of Medical Sciences, Tehran, Iran*

**Keywords:** Milan System for Reporting Salivary Gland Cytopathology, Fine needle aspiration, Risk of malignancy, Salivary gland

## Abstract

**Background & Objective:**

Use of fine needle aspiration cytology (FNAC) in the diagnosis of salivary gland neoplasms is controversial due to the diverse morphologic patterns and overlapping features between benign and malignant lesions. The Milan system has been introduced to report salivary gland cytopathology. The present study aimed to reclassify salivary gland lesions according to the Milan system for reporting salivary gland cytopathology (MSRSGC) to determine the risk of malignancy (ROM) and to estimate the diagnostic accuracy of the Milan system.

**Methods:**

In this retrospective cohort study, 136 salivary gland fine needle aspiration cytology samples taken from patients referred to Imam Khomeini and Amir-Aalam Hospital, Tehran, Iran, from 2016 to 2021, were retrieved along with the histopathological follow-up. Cytology smears were reviewed and reclassified based on MSRSGC. In addition, sensitivity, specificity, positive predictive value (PPV), negative predictive value (NPV), and diagnostic accuracy were calculated.

**Results:**

ROM for each category was 26.7% for non-diagnostic, 12.5% for non-neoplastic, 40% for atypia of undetermined significance (AUS), 0 for benign neoplasm, 0 for salivary gland neoplasm with uncertain malignant potential (SUMP), 100% for suspicious for malignancy, and 100% for malignant group. Sensitivity, specificity, PPV, and NPV in differentiating benign from malignant neoplasms based on MSRSGC were 75.9%, 100%, 100%, and 93.8%, respectively. Diagnostic accuracy was calculated as 94.8%.

**Conclusion:**

MSRSGC may be associated with a high accuracy in differentiation of benign from malignant salivary gland neoplasms, indicating its potential value as an effective classification system for reporting salivary gland cytology. The ROM for cytological categories except SUMP can be almost similar to that suggested by MSRSGC.

## Introduction

Salivary gland tumors account for less than 3% of all head and neck tumors, but they exhibit a wide morphological diversity; even a single type of tumor can have diverse structural patterns (1). Because most of these tumors are benign, it is important to rule out malignancy before surgery to ensure proper treatment (2). Fine needle aspiration cytology (FNAC) is a standard method for diagnosis of salivary gland tumors before surgery (3). This diagnostic procedure is safe, quick, less invasive, cost-effective, and highly accurate, with a low risk of side effects (4). FNAC provides valuable information for differentiating a neoplastic lesion from a non-neoplastic one, thereby determining whether a lesion is benign or malignant. This information is crucial for surgeons in planning appropriate treatments (5-8). However, the accuracy of salivary gland FNAC depends on several factors, including aspiration technique, cytological preparation, intra-tumor heterogeneity, and experience of the cytopathologists (9). The diversity of salivary gland tumors, with significant overlapping in cytomorphology and their rarity, presents a significant challenge in cytological interpretation (10). Thus, this may result in uncertainty in diagnosis for pathologists and decision-making challenges for clinicians (9). Therefore, the American Society of Cytopathology and the International Academy of Cytology have recommended the Milan System for Reporting Salivary Gland Cytopathology (MSRSGC) as a standardized classification system (11). MSRSGC categorizes salivary gland lesions into six diagnostic categories and provides the risk of malignancy (ROM) and clinical management recommendations for each category (12, 13). Given the novelty of this system, global experience with its use is necessary to fully understand its strengths and potential challenges (4, 9). In addition, no study on the effectiveness of the Milan reporting system for salivary gland cytology in Iran has been reported yet. Hence, this study was aimed to reclassify salivary gland lesions according to the MSRSGC to determine ROM and estimate diagnostic accuracy of this system.

## Materials and Methods

This retrospective study was conducted on the cytological reports of fine needle aspiration biopsy (FNAB) specimens of salivary glands taken at Imam Khomeini and Amir Alam hospitals in Tehran, Iran, between 2016 and 2020. The primary cytology slides of these patients were retrieved from the archives of the Department of Anatomical Pathology at our institution. Then using a light microscope, the slides were reviewed by two experienced pathologists who were unaware of the final histopathological diagnosis of the lesion and reclassified based on the criteria of the Milan reporting system for salivary gland cytology reporting. The slides were placed into seven designated categories using this system. If the two pathologists provided different reports on the same case, they reviewed the slides together to reach a consensus and provide a single diagnosis, considered the ‘gold standard.’ In addition, the histopathological diagnosis of the cases that did not align with the cytology report was also reviewed to confirm the final diagnosis. The risk of malignancy (ROM) was then determined in each specified category in the Milan classification system. Finally, this method's sensitivity, specificity, positive predictive value (PPV), negative predictive value (NPV), and accuracy were determined using the following formulas.

Sensitivity=True positive/True positive + false negative

Specificity= True negative/true negative + false positive

Positive predictive value=true positive/cases with a positive test

Negative predictive value=true negative/cases with a negative test

Accuracy=True positive + true negative/true positive and false positive + true negative and false negative

**Table 1:** Correlation between type of the tumor and age, gender, and site of involvement

	Benign	Malignant	Total	P-value
Age, mean ±SD	44.22 14.63	56.17 18.17	46.77 ± 16.14	**0.001**
Gender, n (%) Male Female	54 (72) 53 (86.9)	21 (28) 8 (13.1)	75 (55.1) 61 (44.9)	**0.035**
Tumor site, n (%) Parotid Submandibular	99 (78.6) 8 (80)	27 (21.4) 2 (20)	126 (92.6) 10 (7.4)	0.915

## Results

A total of 136 cases with final histopathological diagnosis and cytology slides were included in this study. There were 75 males (55.1%) and 61 females (44.9%). The mean age of all patients was 46.77±16.14 years (range 9-84 years). The mean age of males was significantly higher than that of females (50.36 vs 42.36 years, *P*=0.008). Of the 136 cases, 126 (92.6%) occurred in the parotid gland and 10 (7.4%) in the submandibular gland. The mean age for malignant neoplasms was higher than that of benign neoplasms (56.17 vs 44.17 years, *P*=0.001). The incidence of malignant neoplasms was higher in males than in females (28% vs 13.1%, *P*=0.035). There was no statistically significant relationship between tumor location and malignancy (*P*=0.915) (Table 1).

A total of 136 cases were reclassified based on the MSRSGC as follows: benign neoplasm, 82 (60.3%); nondiagnostic, 15 (11%); malignant, 13 (9.6%); suspicious for malignancy, 9 (6.6%); nonneoplastic, 8 (5.9%); AUS, 5 (3.7%); and SUMP, 4 (2.9%). According to the final histopathological examination, the most common salivary gland neoplasms were pleomorphic adenoma (67 cases,49.3%), followed by Warthin tumor (19 cases, 14%); mucoepidermoid carcinoma (9 cases, 6.6%); and squamous cell carcinoma (6 cases, 4.4%) (Table 2).

Based on the final histopathological diagnosis following surgery, 107 cases (78.7%) were found to be benign, while 29 (21.3%) cases were malignant. The ROM and risk of neoplasm (RON) by Milan category are suggested in Table 3. There were no false-positive diagnoses, whereas the false-negative rate was 5.1%. Based on the results, the sensitivity, specificity, PPV, and NPV, and the overall accuracy using MSRSGC were 75.9%, 100%, 93.8%, and 94.8%, respectively (Table4).

**Table 2:** Milan system categorical diagnoses correlation with the final surgical diagnoses

Histologic diagnosis	Categories of the Milan System						
	Non-diagnostic, n	Non-neoplastic, n	AUS, n	Benign neoplasm, n	SUMP, n	Suspicious for malignancy, n	Malignant, n
Pleomorphic adenoma	4			60	3		
Spindle cell sarcoma	2						
Chronic sialadenitis	2	1	1				
Normal salivary gland	2			1			
Warthin’s tumor	1			17	1		
Mucoepidermoid carcinoma	1		1			4	3
Adenoid cystic carcinoma	1						2
Lymphoid hyperplasia	1	2					
Lymphoepithelial cyst	1	1	1	1			
Salivary duct cyst		2					
Hemangioma		1					
Squamous cell carcinoma		1				3	2
Basal cell adenoma			1	1			
Lymphoma			1			1	1
Oncocytoma				1			
Myoepithelioma				1			
Salivary duct carcinoma						1	3
Malignant melanoma							2

Abbreviations: N, number; AUS, atypia of undetermined significance; SUMP, salivary gland neoplasm of uncertain malignant potential

**Table 3:** Risk of neoplasm and malignancy associated with MSRSGC

Diagnostic category	No.	RON (%)	ROM (%)
Non-diagnostic	15	60	26.7
Non-neoplastic	8	25	12.5
AUS	5	60	40
Benign neoplasm	82	97.5	0
SUMP	4	100	0
Suspicious of malignancy	8	100	100
Malignant	13	100	100

Abbreviations: ROM, risk of malignancy; RON, risk of neoplasm; AUS, atypia of undetermined significance; SUMP, salivary gland neoplasm of uncertain malignant potential

**Table 4:** Diagnostic performance of the fine needle aspiration cytology of salivary gland tumors (n=136)

Parameter	Values (%)
Sensitivity	75.9
Specificity	100
Positive predictive value	100
Negative predictive value	93.8
Accuracy	94.8

**Fig. 1:**
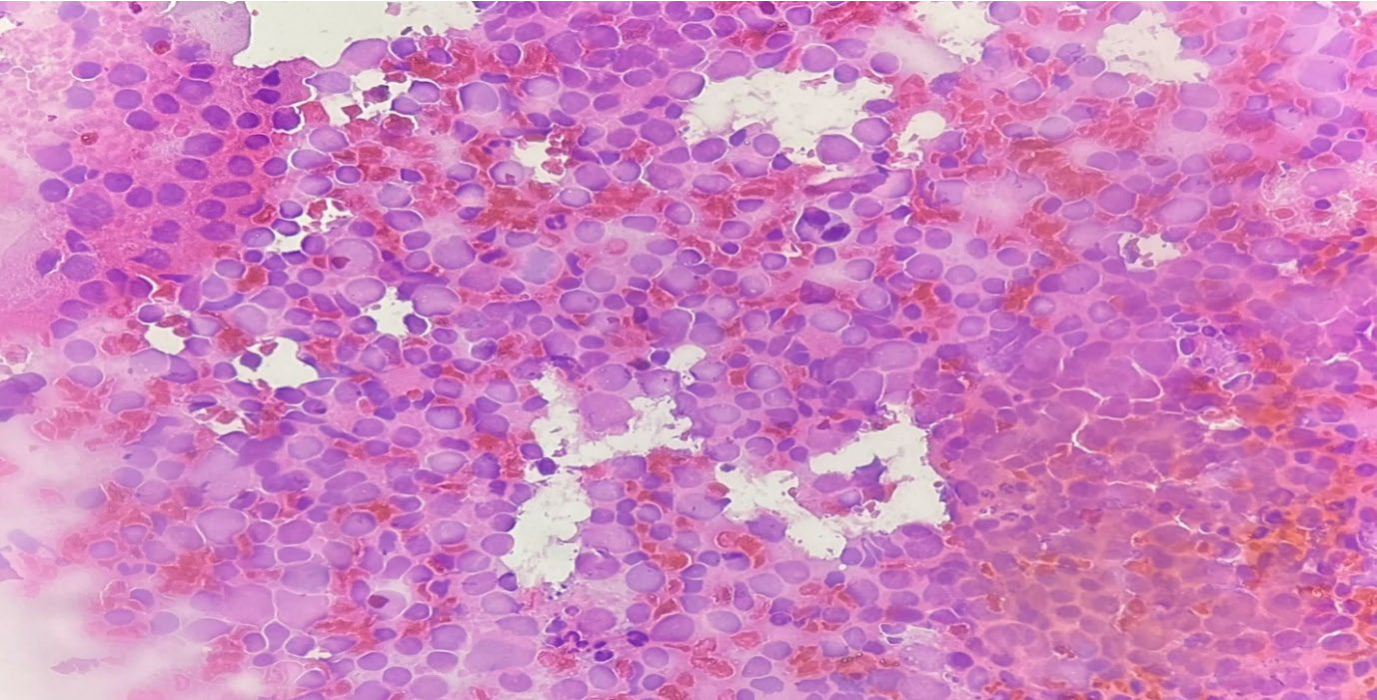
Fine needle aspiration of the parotid gland lymph nodes in an 80-year-old male patient showing abundant mixed population of lymphocyte. A reactive lymph node but low-grade lymphoproliferative disorders cannot be entirely excluded. The lesion was classified as AUS (original magnification ×40).

**Fig. 2:**
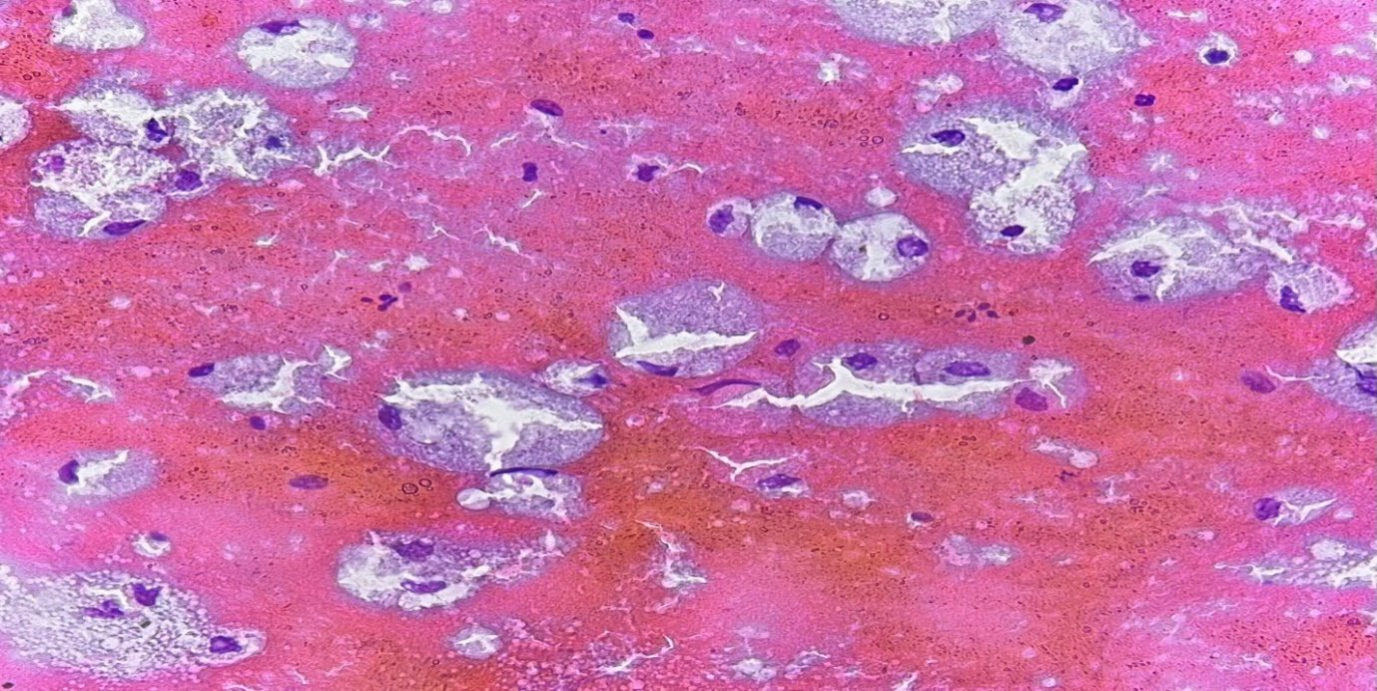
Fine needle aspiration of the parotid gland mass in a 34-year-old female showing cystic content with abundant foamy macrophages and scattered epithelial cells. The lesion was classified as AUS (original magnification ×40).

**Fig. 3:**
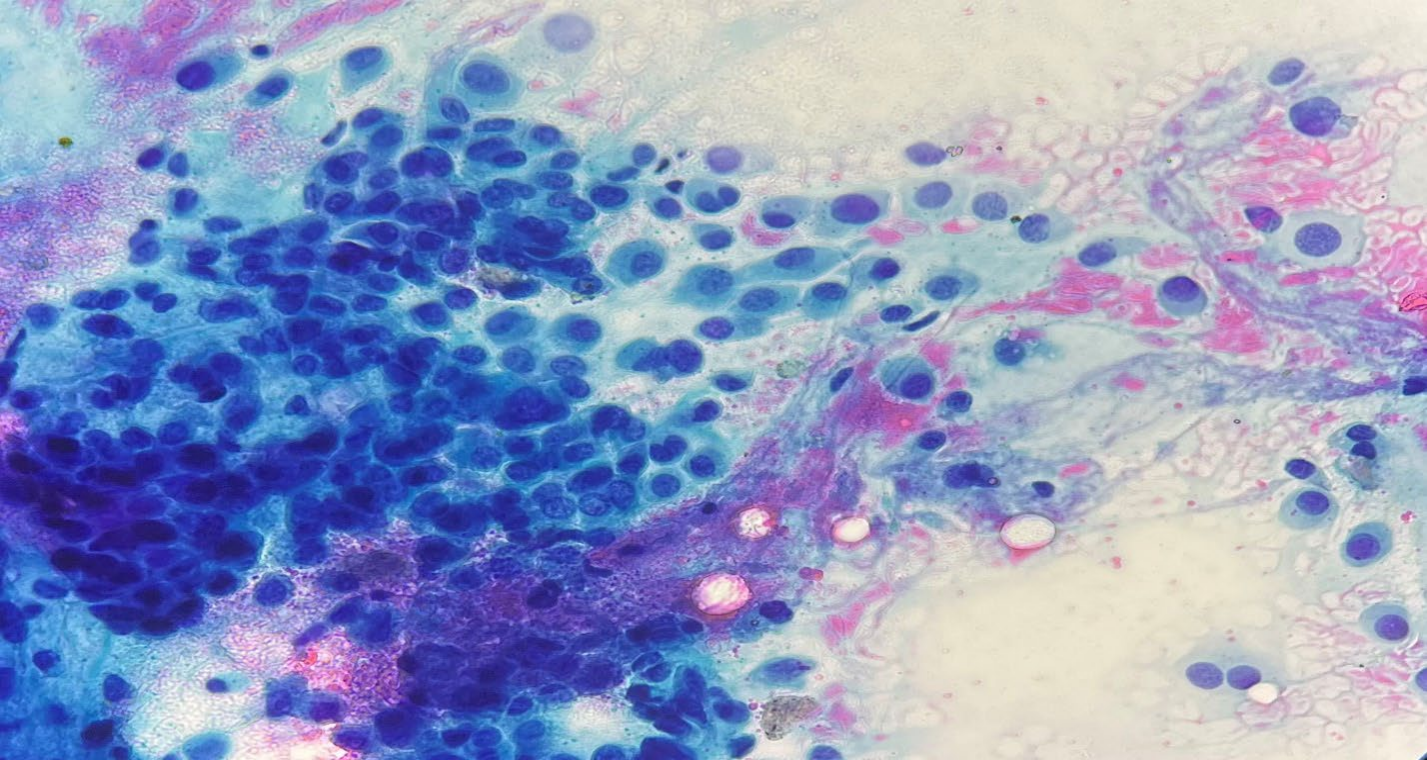
Fine needle aspiration of the parotid gland mass in a 56-year-old female showing a cellular smear composed of polygonal myoepithelial cells in a blue-gray background composed of chondromyxoid matrix with a fibrillary feature. The lesion was classified as a benign neoplasm such as pleomorphic adenoma (original magnification ×40).

## Discussion

MSRSGC aims to establish a standardized reporting system for salivary gland cytopathology. This leads to an effective communication between pathologists and clinicians for correlating cytologic and histologic findings in the cases and facilitating data evaluation and comparison across different institutions (14-16). To standardize this system, studies must be conducted in various medical centers. Hence, the present study demonstrates effectiveness of the MSRSGC in reporting cytology cases of salivary gland lesions for the first time in Iran.

### Nondiagnostic 

In the current study, there were 15 cases in the non-diagnostic category. The non-diagnostic category included cases with quantitatively or qualitatively insufficient material for cytopathological diagnosis. According to Faquin *et al.* (12), the rate of all salivary glands FNACs classified in this category is less than 10%. Studies by Savant *et al.* (10), Song *et al.* (9), and Velaz-Toress *et al.* (17) demonstrated that the frequencies of this diagnostic subgroup were 9.2%, 13.5%, and 16.7%, respectively. Meanwhile, studies conducted in Brazil (18) and Jordan (19) reported frequencies of 19% and 14%, respectively. However, discrepancies in the results across studies can be attributed to factors such as the expertise of the individual performing the sampling and differences in the nature of the tumors. In this study, the non-diagnostic category had 26.7% ROM and 60% RON. In the Millan system, ROM was reported as 25% and in the range of 0-67% for this category, which is similar to the results of our study (12). However, different studies have reported variable ROM for the nondiagnostic category. Savant *et al.* (10) showed no risk of malignancy (0%) and 44.4% RON. A ROM of 33.3% was reported by Jaber et al. (19). Also, Kala et al. (20) study in India and Leite *et al.* (18) study in Brazil found ROM of 25% and 19%, respectively, which relatively similar to the results of our study. In our study, pleomorphic adenoma was the most frequently diagnosed condition (26.7%) and was likely missed due to inadequate sampling and insufficient cellularity. In addition, inflammatory and non-neoplastic lesions, such as chronic sialadenitis, lymph node hyperplasia, lymphoepithelial cysts, and normal salivary glands, accounted for approximately 40% of the cases in the non-diagnostic subgroup. However, 4 malignant neoplasms, including 2 cases of spindle cell sarcoma, 1 case of mucoepidermoid carcinoma, and 1 case of adenoid cystic carcinoma, were also classified in this category. Inadequate sampling may have occurred due to various reasons, such as sampling from cystic degeneration areas, small lesion size, difficult lesion access, and sampling errors. Nevertheless, according to MSRSGC, it is recommended to repeat sampling of the lesion and consider clinical and radiological correlation in these cases (12).

### Non-neoplastic

In our study, 8 cases were classified as a nonneoplastic category of MSRSGC. The ROM and RON for this category were 12.5% and 25%, respectively. The ROM found in this study was 10% and 10.2%, respectively, as recommended by the MSRSGC in the literature (11, 15). In some studies, the ROM in this category is as low as zero (10, 18, 21). However, other research has reported higher rates of malignancy, ranging from 18% to 20% (9, 17). Specifically, studies by Rossi et al. (16) and Rohilla et al. (22) have shown varying levels of ROM in non-neoplastic cases. In contrast, studies by Viswanathan et al. (23) in America and Wu et al. (24) in Taiwan have reported slightly lower ROM, around 7%- 9%. In the present study, 2 cases were classified as non-neoplastic on histopathology, including a case of hemangioma and a case of squamous cell carcinoma. Hemangioma and lipoma may usually go undiagnosed due to their hypocellular nature. Well-differentiated squamous cell carcinoma, especially with cystic degeneration in the head and neck area, is another dilemma for the pathologist, which can mimic branchial cleft cyst or epidermal inclusion cyst. In such a situation, clinicopathological correlation for proper classification is crucial. According to the MSRSGC, the clinical management suggested for this category is clinical follow-up and radiological correlation (12)

### AUS

In this study, 5 cases were classified as AUS consisting of 2 cases of non-neoplastic lesions (chronic sialadenitis and lymphoepithelial cyst), one benign neoplasm (basal cell adenoma), and 2 cases of malignant neoplasm (lymphoma and mucoepidermoid carcinoma). In this study, the AUS category had 40% ROM and 60% RON. Our ROM was higher than that recommended by MSRSGC (20%) (12). This may be attributed to the small number of cases included in this category in our study. In a study performed in Brazil, the AUS category's ROM was 40%, similar to our study (18). In other studies, performed in America and Jordan, ROM was reported in approximately 33% of cases (10, 19). Furthermore, studies in Taiwan and Spain found that 37.5% and 46% of the cases categorized as AUS were ultimately diagnosed as malignant neoplasms (21, 24). The variation in results among different studies can be attributed to several factors, such as heterogeneity of the samples, some inherent characteristics of the aspirated mass, and technical factors related to the sample preparation, which make cytological diagnosis and accurate classification challenging. In our study, 3 cases including 1 case of chronic sialadenitis, 1 case of lymphoepithelial cyst, and 1 case of marginal zone lymphoma related to lymphoid infiltration (Figure 1). In addition, a case of low-grade mucoepidermoid carcinoma was included in the AUS category, which mainly contained cystic components, foamy macrophages, and a small number of epithelial cells, cytologically. The features seen were probably due to cystic degeneration in the lesion (Figure 2). According to the MSRSGC, salivary gland lesions with a cytological diagnosis of AUS should undergo re-FNA or surgery according to the clinical and radiological evidence (12).

### Benign Neoplasm

Benign neoplasms were the most common (82 cases) cytological subtype observed in our study. This finding aligns with the results reported in other studies. In the study by Savant *et al.* (10), 59% of the cases were classified in the benign neoplasm category. Lower ROMs were reported by Velez-Toress *et al.* (17), and Leite *et al.* (18), who calculated ROMs were 49.4% and 40%, respectively. The ROM and RON for this category were 0% and 97.5%, respectively. The ROM obtained in our study for the benign neoplasm category is similar to the value recommended by the MSRSGC (less than 5%) (11). In line with our study findings, both the Gaikwad study in India and the Jaber study in Jordan reported a ROM of zero in the benign neoplasms category (19, 25). In the Savant study in the United States, the ROM was approximately 0.8%, and the RON was 100%, which is close to the results of our study (10). Other studies have reported the ROM to be less than 5% for this category (9, 17, 20, 24, 26). In the present study, about 95% of the cases in this category comprised pleomorphic adenoma (Figure 3) and Warthin tumor. In addition, one case of basal cell adenoma, one case of oncocytoma, and one case of myoepithelioma were also classified in this category. Only 2 non-neoplastic cases, including 1 case of lymphoepithelial cyst and one case of normal salivary glands, were misclassified in this category. The high diagnostic accuracy reported in various studies for this category may be attributed to the comprehensive descriptions of the cytomorphologic characteristics of benign salivary gland tumors found in the literature. The low ROM in neoplasms of this category enables clinicians to perform appropriate surgical resection without requiring intraoperative consultation through frozen section. The MSRSGC also recommends conservative surgery or clinical follow-up for this category (12).

### SUMP

In the present study, 4 cases were classified as SUMP. This category comprises specimens that demonstrate neoplastic characteristics based on their cytological features, but it is difficult to distinguish between a benign or malignant nature. In the present study, three samples of pleomorphic adenoma and one sample of Warthin’s tumor were included in this category. ROM was zero, and RON was 100% among our studied cases. However, our ROM was below the 35% suggested by MSRSGC (11). Some studies have reported a 100% ROM in the SUMP category (21, 25). However, in the study by Leite *et al.* (18) in Brazil, the ROM was 13.3%. Nevertheless, in most studies, the ROM for the SUMP category has been in the range of 30-50% (9, 10, 20, 24). The variation in reported ROM for the SUMP category could be attributed to the small sample size and heterogeneity of the included lesions within this category. The MSRSGC recommends conservative surgery for tumors in the SUMP category. The type of surgery should be determined following preoperative imaging and intraoperative consultation to assess tumor spread, margins, and lymph node involvement (12).

### Suspicious of Malignancy

In the current study, 8 cases were classified as suspicious for malignancy. The estimated ROM for this category was 100%, which was higher than the 60% ROMs proposed by MSRSGC (12). Several studies, including those conducted by Savant (10) in America, Wu *et al.* (24) in Taiwan, Jaber *et al.* (19) in Jordan, and Gaikwad *et al.* (25) in India, reported a 100% ROM in the SUMP category, which aligns with the findings of our study. Additionally, Viswanathan *et al.* (23) reported a 92.9% ROM. Other studies conducted in America and Brazil also found the ROM in this category to be higher than 70% (9, 17, 18). In our study, despite the high estimated ROM for this category, there were no false positive results. The most common types of malignancies in this category were mucoepidermoid carcinoma (44.4%) and squamous cell carcinoma (33.3%), with one case diagnosed as diffuse large B-cell lymphoma. The MSRSGC recommends surgery for cases in this category (12). However, a study suggested that the cytology report should specify the type of suspected malignancy, especially for lymphoproliferative lesions, to improve treatment management (10).

### Malignant 

In the present study, the malignant category comprised 13 cases of the FNA samples. The ROM for this category was 100%, consistent with several studies (10, 18, 21, 25, 26), although slightly higher than the suggested ROM by MSRSGC (12). In some other studies, the ROM was 97%- 98.5%. This category's most common histological findings included mucoepidermoid carcinoma and salivary duct carcinoma. Notably, there were no false positive cases in the malignant cytological category in the study. The MSRSGC has recommended surgery for cases classified as malignant (12).

In the present study, the most common malignancies were mucoepidermoid carcinoma, with 9 cases (6.6%), followed by squamous-cell carcinoma, with 6 cases (4.4%). The same results were reported by Rezvani *et al.* (27), Wu *et al.* (24), and Rossi *et al.* (16), while in the study of Viswanathan *et al.* (23), lymphoma was found to be the most common malignancy. 

Our results showed that the diagnostic sensitivity, specificity, PPV, NPV, and accuracy of salivary gland MSRSGC were 75.9%, 100%, 100%, 93.8%, and 94.8%, respectively. The values are comparable to previous studies (10, 18, 22, 24, 25). 

## Conclusion

Our study confirms a possible high accuracy of MSRSGC in differentiating between benign and malignant salivary gland neoplasms, indicating its utility as a potential effective classification system for reporting salivary gland cytology. The ROM for cytological categories except SUMP was similar to that suggested by MSRSGC and other studies conducted in this field. Given the small number of cases in the SUMP category in the present study, further studies are necessary to establish definitive conclusions. 

## References

[1] Galdirs TM, Kappler M, Reich W, Eckert AW (2019). Current aspects of salivary gland tumors - a systematic review of the literature. GMS Interdiscip Plast Reconstr Surg DGPW..

[2] Eveson JW, Cawson RA (1985). Salivary gland tumours A review of 2410 cases with particular reference to histological types, site, age and sex distribution. J Pathol.

[3] Vallonthaiel AG, Kaushal S, Jangir H, Rajendran HK (2018). Application of the Milan System for Risk Stratification and Its Comparison with a Previous Reporting System of Parotid Gland Cytopathology in a Tertiary Care Centre. Acta Cytol.

[4] Karuna V, Gupta P, Rathi M, Grover K, Nigam JS, Verma NJIJoP (2019). Effectuation to Cognize malignancy risk and accuracy of fine needle aspiration cytology in salivary gland using "Milan System for Reporting Salivary Gland Cytopathology": A 2 years retrospective study in academic institution. Indian Journal of Pathology and Microbiology.

[5] Wang H, Fundakowski C, Khurana JS, Jhala N (2015). Fine-Needle Aspiration Biopsy of Salivary Gland Lesions. Arch Pathol Lab Med.

[6] Goyal S, Sharma S, Diwaker P (2015). Diagnostic role and limitations of FNAC in oral and jaw swellings. Diagn Cytopathol.

[7] Seethala RR, Griffith CC (2016). Molecular Pathology: Predictive, Prognostic, and Diagnostic Markers in Salivary Gland Tumors. Surg Pathol Clin.

[8] Pusztaszeri MP, Garcia JJ, Faquin WC (2016). Salivary gland FNA: New markers and new opportunities for improved diagnosis. Cancer Cytopathol.

[9] Song SJ, Shafique K, Wong LQ, LiVolsi VA, Montone KT, Baloch ZJC (2019). The utility of the Milan System as a risk stratification tool for salivary gland fine needle aspiration cytology specimens. Cytopathology.

[10] Savant D, Jin C, Chau K, Hagan T, Chowdhury M, Koppenhafer J (2019). Risk stratification of salivary gland cytology utilizing the Milan system of classification. Diagn Cytopathol.

[11] Rossi ED, Faquin WCJCC (2018). The Milan System for Reporting Salivary Gland Cytopathology (MSRSGC): an international effort toward improved patient care-when the roots might be inspired by Leonardo da Vinci. Cancer Cytopathology.

[12] Faquin WC, Rossi ED, Baloch Z, Barkan GA, Foschini MP, Kurtycz DF (2018). The Milan system for reporting salivary gland cytopathology.

[13] Rossi ED, Faquin WC, Baloch Z, Barkan GA, Foschini MP, Pusztaszeri M (2017). The Milan System for Reporting Salivary Gland Cytopathology: Analysis and suggestions of initial survey. Cancer Cytopathol.

[14] Schmidt RL, Hall BJ, Wilson AR, Layfield LJ (2011). A systematic review and meta-analysis of the diagnostic accuracy of fine-needle aspiration cytology for parotid gland lesions. Am J Clin Pathol.

[15] Wei S, Layfield LJ, LiVolsi VA, Montone KT, Baloch ZW (2017). Reporting of fine needle aspiration (FNA) specimens of salivary gland lesions: A comprehensive review. Diagn Cytopathol.

[16] Rossi ED, Wong LQ, Bizzarro T, Petrone G, Mule A, Fadda G (2016). The impact of FNAC in the management of salivary gland lesions: Institutional experiences leading to a risk-based classification scheme. Cancer Cytopathol.

[17] Velez Torres JM, Tjendra Y, Zuo Y, Garcia-Buitrago M, Jorda M, Kerr DA (2022). Application of the Milan System for Reporting Salivary Gland Cytopathology: A Single Institutional Experience of 354 Cases with Cytologic-Histologic Correlation. Acta Cytol.

[18] Leite AA, Vargas PA, Dos Santos Silva AR, Galvis MM, de Sa RS, Lopes Pinto CA (2020). Retrospective application of the Milan System for reporting salivary gland cytopathology: A Cancer Center experience. Diagn Cytopathol.

[19] Jaber OI, Shawash SI (2021). Retrospective implementation of the Milan system for reporting salivary gland cytopathology; a review of 5 years in a specialized cancer center. Diagn Cytopathol.

[20] Kala C, Kala S, Khan L (2019). Milan System for Reporting Salivary Gland Cytopathology: An Experience with the Implication for Risk of Malignancy. J Cytol.

[21] Val-Bernal JF, Martino M, Marcos S, Yllera E, Garcia-Montesinos B (2020). Fine-needle aspiration cytology in the diagnosis of salivary gland lesions The role of the Milan system for reporting cytopathology. Acta Otorrinolaringol Esp (Engl Ed).

[22] Rohilla M, Singh P, Rajwanshi A, Gupta N, Srinivasan R, Dey P (2017). Three‐year cytohistological correlation of salivary gland FNA cytology at a tertiary center with the application of the Milan system for risk stratification. Cancer Cytopathol.

[23] Viswanathan K, Sung S, Scognamiglio T, Yang GCH, Siddiqui MT, Rao RA (2018). The role of the Milan System for Reporting Salivary Gland Cytopathology: A 5-year institutional experience. Cancer Cytopathol.

[24] Wu HH, Alruwaii F, Zeng BR, Cramer HM, Lai CR, Hang JF (2019). Application of the Milan System for Reporting Salivary Gland Cytopathology: A Retrospective 12-Year Bi-institutional Study. Am J Clin Pathol.

[25] Gaikwad VP, Anupriya C, Naik LPJJoC (2020). Milan system for reporting salivary gland cytopathology-An experience from Western Indian Population. Journal of Cytology.

[26] Chen YA, Wu CY, Yang CS (2019). Application of the Milan System for Reporting Salivary Gland Cytopathology: A retrospective study in a tertiary institute. Diagn Cytopathol.

[27] Rezvani G, Yazdani Bioki F, Khatami R, Keramat H, Moadabi A (2015). Reliability of Fine Needle Aspiration Cytology for Salivary Gland Lesions. J Iran Dent Assoc.

